# Plasma IL-17A is increased in patients with critical MIS-C and associated to in-hospital mortality

**DOI:** 10.3389/fimmu.2024.1485009

**Published:** 2025-01-27

**Authors:** Emmerson C. F. de Farias, Luciana M. P. P. do Nascimento, Manoel J. C. Pavão Junior, Dalila C. A. Pavão, Ana P. S. Pinheiro, Andreza H. O. Pinheiro, Marília C. B. Alves, Kíssila M. M. M. Ferraro, Larisse F. Q. Aires, Luana G. Dias, Mayara M. M. Machado, Michaelle J. D. Serrão, Raphaella R. Gomes, Sara M. P. de Moraes, Gabriela C. L. Pontes, Railana D. F. P. Carvalho, Cristiane T. C. Silva, Carla M. A. das Neves, Joyce C. L. dos Santos, Adriana M. B. de Sousa, Leda L. da Silva, Mary L. F. M. F. de Mello, Patricia B. Carvalho, Renata de B. Braga, Kathia de O. Harada, Maria C. A. Justino, Iran B. Costa, Igor Brasil-Costa, Marta C. Monteiro, Gleice Clemente, Maria Teresa Terreri

**Affiliations:** ^1^ Division of Pediatric Intensive Care, Department of Pediatrics, Fundação Santa Casa de Misericórdia do Pará, Belém, Brazil; ^2^ Division of Pediatric Intensive Care, Departament of Pediatrics, Fundação Hospital das Clínicas Gaspar Viana, Belém, Brazil; ^3^ Clinical Research Unit, Health Surveillance Secretariat, Brazilian Ministry of Health, Instituto Evandro Chagas, Ananindeua, Brazil; ^4^ Immunology Laboratory, Virology Unit, Instituto Evandro Chagas, Ananindeua, Brazil; ^5^ Pharmaceutical Science Post-Graduation Program and Neuroscience and Cell Biology Graduate Program, Health Science Institute, Federal University of Pará/UFPA, Belém, Brazil; ^6^ Division of Pediatric Rheumatology, Department of Pediatrics, Universidade Federal de São Paulo, São Paulo, Brazil

**Keywords:** COVID-19, cytokines, mortality, oxidative stress, pediatric intensive care unit, post-acute COVID-19 syndrome

## Abstract

**Background:**

Multisystem inflammatory syndrome in children (MIS-C) is a rare and severe post-COVID-19 complication with multiple phenotypes.

**Objectives:**

The aim of this study is to study inflammatory biomarkers (cytokines and oxidative stress) in critical MIS-C patients and to observe if there is association between these biomarkers and mortality.

**Methods:**

A single-center prospective study enrolled patients with MIS-C (with positive molecular test), aged between 1 month and 18 years of age. Data was collected from 20 pediatric intensive care unit (PICU)’s bed. Inflammatory biomarkers (cytokines and oxidative stress markers) were performed on day 1 and 3 after hospitalization. Survival rate was calculated, and Kaplan-Meier curves were plotted. Univariate and multivariate Cox regression analyses were conducted. The ROC (Receiver Operating Characteristic) curve analysis was performed.

**Results and conclusions:**

A total of 41 patients out of 109 patients admitted at PICU with suspected MIS-C during the study period were included, of which 33 (80.5%) were male, 9 (22%) were under one year old, and 30 (73.2%) presented comorbidities. Among them, 16 (39%) did not survive. The mean survival time was shorter in patients with higher levels of IL-17A (≥ 19.71 pg/mL) on day 1 (115 vs 323 days, p = 0.004). Higher levels of IL-17A on day 1 were associated with mortality in both the crude model (HR 1.03, CI95% 1.004-1.057, p = 0.022) and the adjusted model (HR 1.043, CI95% 1.013-1.075, p = 0.012). ROC analysis revealed a cut-off value for the IL-17A of 14.32 pg/ml. The other immunological and inflammatory markers did not demonstrate an association with survival (p>0.05). Our findings suggest that patients with high levels of IL-17A are at greater risk for death.

## Introduction

At the onset of the coronavirus disease 2019 (COVID-19) pandemic, a severe multisystem inflammatory syndrome in children (MIS-C) emerged in various countries across Europe and North America (1–4). MIS-C is typified by systemic inflammation, with clinical, epidemiological, or microbiological indicators of exposure to severe acute respiratory syndrome coronavirus 2 (SARS-CoV-2). Presently, it is considered a rare yet severe complication post-COVID-19, presenting with multiple clinical phenotypes (4). Consequently, early recognition, intervention, and admission to a pediatric intensive care unit (PICU) are typically necessary (1–5).

In both COVID-19 and MIS-C, as seen in other chronic infections, there is a notable dysregulation involving hyperactivation of CD4^+^ T lymphocytes, potentially leading to the exhaustion of CD8^+^ T lymphocytes. This dysregulation suggests a reduction in antiviral immunity, which may exacerbate the clinical severity of MIS-C, particularly in children under the age of two (6). A distinct immune response is noted between COVID-19 and MIS-C, characterized by a persistent elevation in pro-inflammatory cytokines such as IL-1β, IL-2, IL-6, IFN-α, IL-10, IL-17, granulocyte-macrophage colony-stimulating factor (GM-CSF), and high-mobility group box 1 (HMGB1) among MIS-C patients (6–12).

Respiratory viral infections, including SARS-CoV-2, can instigate genomic damage by provoking excessive cytokine release, elevating inflammatory levels that destabilize immune response (6, 7). This inflammatory cascade often coincides with increased intracellular levels of reactive oxygen species (ROS), causing oxidative damage to lipids, proteins, and DNA—a condition termed oxidative stress (OS) (13, 14). Prior research has shown that such cytokine release is amplified during COVID-19, potentially resulting in acute lung injury and, in severe cases, mortality (6–12). Elevated OS has also been suggested as a contributory factor in the disease’s pathogenesis (13–18).

Emerging research underscores the significant role of OS and cytokine dysregulation in the pathophysiology of MIS-C associated with COVID-19. Elevated ROS levels have been associated with cellular damage in COVID-19 cases, with oxidative damage to lipids, proteins, and DNA potentially leading to organ dysfunction and severe outcomes (12, 13, 17). In MIS-C, specific oxidative stress biomarkers—such as catalase (CAT), malondialdehyde (MDA), and trolox equivalent antioxidant capacity (TEAC)—have shown variable levels, some correlating with disease severity and mortality (15, 16, 18). These biomarkers reflect oxidative damage as well as the body’s compensatory antioxidant mechanisms, offering valuable insights into the severity of MIS-C (12–18).

However, current studies on ROS and cytokine profiles in MIS-C remain limited. Most available studies explore antioxidant therapies in non-pediatric populations (13, 14), focus on mixed pediatric populations (15–20), or employ experimental murine models (21, 22), while few concentrate specifically on critical MIS-C cases and their association with mortality.

The primary objective of this study is to evaluate inflammatory biomarkers in MIS-C patients admitted to the pediatric intensive care unit (PICU) and to determine their potential as predictors of in-hospital mortality. A secondary objective is to assess the relationship between these biomarkers and the occurrence of cardiogenic shock, as well as PICU length of stay. Additionally, we aim to compare laboratory parameters between days 1 and 3 of hospitalization and to evaluate the accuracy of IL-17A levels in predicting mortality among this patient population.

## Material and methods

### Study design and subjects

This was a single center observational prospective study with a convenience sample consisting of patients diagnosed with MIS-C between one month and 18 years of age, with confirmed SARS-CoV-2 infection (molecular test), and who required hospitalization in PICU. Patients were recruited from a tertiary Service in Belém, state of Pará, Brazil, between May 1, 2020, and December 31, 2023. All patients included in this study fulfilled the MIS-C case definition of World Health Organization (WHO) (4). Patients with similar presentation but with positive microbiology other than SARS-CoV-2 and those with immunosuppression status were excluded. Ethics approvals were obtained from the institutional review board of the Institution. We obtained a waiver of written informed consent to conduct this observational study.

Recognizing the unprecedented challenges posed by MIS-C and the need for comprehensive biomarker analysis, a task force was organized to support both the clinical and research aspects of this study. In practical terms, despite the designation as a single-center study, an informal network of pediatric intensivists in the region collaborated to facilitate active case identification. As MIS-C cases were identified in other local PICUs, the attending physicians promptly contacted the lead investigator to expedite the transfer of patients meeting MIS-C criteria to the primary institution for consistent clinical and laboratory management. This approach ensured a streamlined and uniform protocol for patient assessment and sample collection. The biomarker analyses were conducted within this centralized framework, though specific aspects required external partnerships for optimized processing. The oxidative stress measurements were conducted in collaboration with the Laboratory of *In Vitro* and Microbiology Assays at the Federal University of Pará, and cytokine level assessments were performed at the Evandro Chagas Institute’s Immunology Laboratory. These collaborations aimed to enhance the diagnostic and prognostic understanding of MIS-C, especially considering the notably higher mortality rates observed in our region compared to global data. As for the ethics approval process, the institutional review board approval was obtained solely from the primary hospital, as this institution served as the formal site of data collection, patient care, and biomarker analyses.

The PICU fulfil international criteria (23) for quaternary care levels, characterized by having a high level of resources and access to Mechanical Ventilation (MV), Renal Replacement Therapy (RRT), vasoactive/inotropic medications, and treatments targeted to MIS-C (Intravenous Immunoglobulin [IVIG] and steroids). They also serve as training centers for medical and health professionals.

Patients were splitted into two groups based on the main outcome, i.e., in-hospital mortality up to 28^th^ day: survivors and non-survivors. Presence of cardiogenic shock, critical disease, and length of stay at the Hospital were evaluated. The 28-day mortality endpoint was assessed according to current pattern of outcome measures of clinical studies in order not to achieve an ambiguous measure (24). All outcomes and the worst features were recorded and compared between the 2 groups at admission and on the third day.

### Data collection and definitions

Clinical and laboratory data as well as demographic and epidemiological characteristics were collected from electronic records of the patients in PICU. Intensive support characteristics were also assessed sequentially until the end of hospitalization. The patients presented critical or non-critical disease according to the multiple organ dysfunction syndrome (MODS) but at least two organic dysfunctions should be present (25–28).

The presence of critical illness was determined by the finding of MODS which was defined as the simultaneous occurrence of three or more organ dysfunctions according to previously published criteria (25–28). Some patients in our study were hospitalized in the PICU only for monitoring, and they were not considered for classification in critical illness. The definition and categorization of comorbidities were based on the classification by Feudtner et al., named Pediatric Complex Chronic Conditions (PCCC) (29).

In the current study, circulatory shock was defined by altered levels of consciousness, decreased urine output (< 1 mL/kg/h or 12 mL/m²/h), and lactic acidosis, with arterial hypotension being a late sign of shock in children. Meanwhile, cardiogenic shock was defined by the presence of an ejection fraction less than 55%, as assessed by a pediatric echocardiographer using the Teichholz method, troponin elevation to twice the reference value, and the presence of clinical signs of circulatory shock (30, 31). Severely immunocompromised patients: HIV carriers; patients on immunosuppressive therapy post-solid organ or bone marrow transplant; neutropenic patients (below 500 neutrophils/mm^3^ or below 1000/mm^3^ with a downward trend); patients undergoing chemotherapy in the last 28 days or other immunosuppressive therapies; patients on immunosuppressive dose of corticosteroid (equivalent to 2mg/kg/day of prednisone for more than 2 weeks in those under 10 kg or 20mg/day for more than 2 weeks or 40mg of prednisone for ≥ 10 days in those over 10 kg) (32).

### Analysis of inflammatory biomarkers

Blood samples were drawn by venipuncture from all patients on day 1 and 3 of the admission, before the initiation of immunomodulatory therapy. For complete blood count parameters, 2 mL of blood was taken into in tubes containing ethylenediaminetetraacetic acid (EDTA). The plasma obtained was stored at − 80°C. Next, cytokines and oxidative stress biomarkers were measured.

### Determination of cytokines profile

Plasma levels of seven cytokines (IL-2, IL-4, IL-6, IL-10, TNF, IFN-γ and IL-17A) were analyzed after filtering proteins with measurements > 30% below the detection threshold, by CBA according to the instruction of BD™ Cytometric Bead Array (CBA) Human Th1/Th2/Th17 Cytokine Kit (Becton & Dickinson, CA, USA). Measurement by flow cytometry was performed using a FACSCanto™ II cytometer (Becton & Dickinson, CA, USA). Plasma levels of the respective cytokines were expressed in pg/ml.

### Determination of plasma nitrite levels

The nitrate (NO_3_^−^) present in the serum samples was converted to nitrite (NO_2_^−^) with nitrate reductase, and the nitrite concentration was determined using the Griess method (33). In brief, 100 *μ*L of the supernatant samples were incubated with an equal volume of Griess reagent for 10 min at room temperature. The absorbance was measured on a plate scanner (Spectra Max 250; Molecular Devices, Menlo Park, CA, USA) at 550 nm. The nitrite concentration was determined using a standard curve generated using sodium nitrite (NaNO_2_). Nitrite production was expressed per *μ*M.

### Determination of plasma malondialdehyde concentration

The MDA was used for the reaction of thiobarbituric acid reactive substances (TBARS) performed according to the adapted form (34) of a previously proposed method (35). An aliquot of 1 mL of the reagent (TBA 10 nM) and 0.5 mL of the sample was added to each test tube. Then, the tubes were placed in a water bath at 94°C for 1 h. After this procedure, the samples were cooled under running water for about 15 minutes, and then 4 mL of butyl alcohol was added to each sample. Subsequently, the samples were mixed on a vortex shaker, in order to obtain the maximum extraction of MDA into the organic phase. Finally, the tubes were centrifuged at 2,500 rpm for 10 minutes. A volume of 3 mL of supernatant was pipetted to carry out spectrophotometric reading at 535 mm. Results were expressed in nmol/mL^-1^.

### Determination of trolox equivalent antioxidant capacity

The trolox equivalent antioxidant capacity (TEAC) is a sensitive and reliable marker for detecting *in vivo* oxidative stress markers that may not be detectable through the measurement of a single specific antioxidant (36). TEAC level of the plasma serum samples was measured using a previously developed method (37). In this assay, 7 mM of 2,2-azinobis, 3-ethylbenzothiazoline, 6-sulfonate (ABTS) were incubated with 2.45 mM of potassium persulfate and ABTS-potassium persulfate (1: 0.5, v/v), and the mixture was allowed to stand in the dark at room temperature for 12–16 h before use. For the study, the blue green ABTS+ solution was diluted with ethanol 95% (v/v) until the absorbance reached 0.70 ± 0.02 at 734 nm. Then, 10 *μ*L of the plasma or trolox standard was mixed with 1 mL of ABTS+ solution, and a decrease in absorbance at 734 nm was recorded after 4 min for all samples. The absorbance of the mixture was monitored at 734 nm after 6 min. For the blank, 10 *μ*L of water instead of the sample was used and each sample was measured in triplicate. Total antioxidant potential of plasma serum was expressed as *μ*mol/mL of TEAC and was calculated through a calibration curve plotted with different amounts of trolox (38).

### Determination of catalase activity

Catalase (CAT) activity was determined according to a previously validated method (39). Blood samples were hemolyzed into ice water (1: 3) and then diluted in a Tris based buffer (Tris 1 M/EDTA 5 mM, pH 8.0). To verify the decay of hydrogen peroxide (H_2_O_2_), aliquots of the diluted samples were added to 900 *μ*L of reaction solution (Tris base, H_2_O_2_ 30% and ultrapure water, pH 8) (40). The decrease of H_2_O_2_ concentration was established at *λ* = 240 nm at 25°C for 60 seconds. CAT activity was defined as the activity required to degrade 1 mol of H_2_O_2_ for 60 seconds (pH 8 and 25°C) and was expressed as U/mg protein. The molar extinction coefficient of H_2_O_2_ used for the calculation was 39.4 cm (2)/mol. The enzymatic activity data obtained in CAT assays were normalized by the total protein concentrations, using the commercial kit (Doles, Brazil).

### Determination of glutathione reductase levels

Determination of intracellular GSH levels was based on the ability of GSH to reduce 5,5-dithiobis-2-nitrobenzoic acid (DTNB) to nitrobenzoic acid (TNB), which was quantified by spectrophotometry at 412 nm. The methodology described by Ellman (41) as adapted for this determination, and GSH concentrations, were expressed in µmol/mL. This assay was adapted (42) for use in a microtitre plate using a microplate spectrophotometer system, Spectra MAX 250 (Molecular Devices, Union City, CA, USA).

### Statistical analysis

The cohort characteristics were summarized using median (interquartile range) for continuous variables, and absolute numbers and frequencies (%) for categorical variables. Comparisons between groups were made using χ^2^ or Fisher exact tests for categorical variables, and Mann–Whitney U was used for continuous data. For comparison between day 1 and day 3 the Wilcoxon signed-ranks test was used. Holm-Bonferroni correction for multiple comparisons was employed adjusting the significance level when patients were compared between two groups (survivors and non-survivors).

We used the ROC (Receiver Operating Characteristic) analysis to choose the optimal cut-point associated with mortality outcome. Survival rate in days was calculated, and Kaplan-Meier curves were plotted. We performed a univariate and multivariate Cox regression “time - to first - event” analysis to determine risk factors for survival. In the regression model we adjusted for a risk of mortality using age under one year of age and presence of comorbidity simultaneously. For these analyses the adopted significance level was set at two-sided p <0.05. The ROC curve analysis was performed to determine the effectiveness of IL-17A as a biomarker to predict the likelihood of death in patients. Data analyses were conducted using SPSS (Statistical Package for the Social Sciences, Chicago, IL) version 28.0.

## Results

### Cohort description

**Figure 1:**
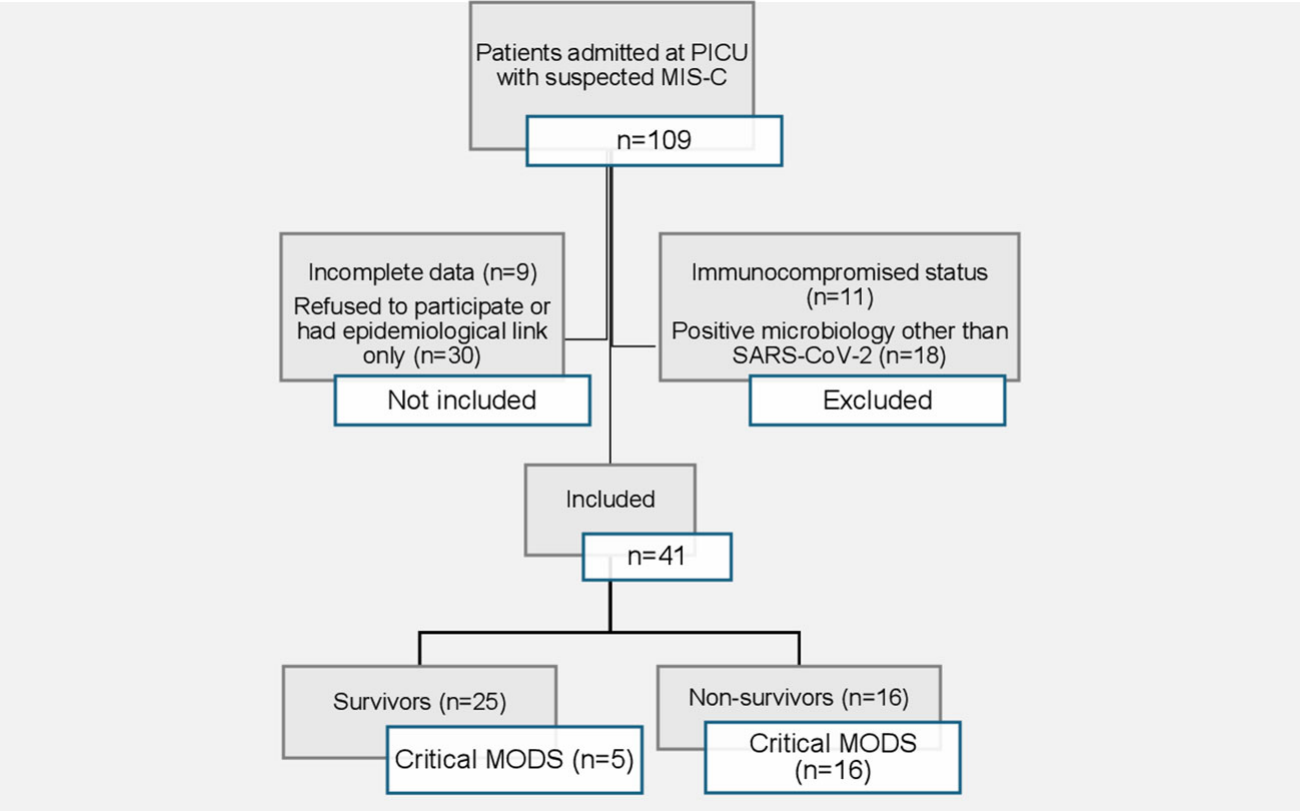
Fluxogram describing the study selection.

There were 109 patients admitted at PICU with suspected MIS-C during the study period. A total of 41 patients (median age of 55 months) were included (**Figure 1**). Thirty-three (80.5%) were male, nine (22%) were up to one year of age, and 30 (73.2%) presented PCCC. The median time from SARS-CoV-2 exposure to symptom onset was 23 days (IQR: 15-34, days). Critical disease and presence of more than three organ dysfunctions occurred in 21 (52.1%) and 26 (63.4%) patients, respectively. Invasive mechanical ventilation was required in 22 (53.7%), and deaths occurred in 16 (39%) patients (**Table 1**).

**Table 1:** Baseline characteristics of the cohort.

Characteristics	Hospitalized children with MIS-C (n=41)
Demographics and epidemiological	
Age in months, median (IQR)	55 (16-111.8)
Children under 1 year old, n (%)	9 (22)
Male, n (%)	33 (80.5)
Ethnicity: Brazilian Pardo, n (%)	36 (87.8)
Pediatric complex chronic conditions, n (%)	30 (73.2)
Main Pediatric complex chronic conditions: Neurologic & neuromuscular, n (%)	7 (23.3)
BMI-for-age Z-scores classification for children under 5 years old: severely underweight, n (%)*	7 (28)
BMI-for-age Z-scores classification for children over 5 years old: moderately underweight, n (%)**	5 (31.3)
Time from exposure to symptom onset in days, median (IQR)	23 (15-34)
Duration of fever in days, median (IQR)	12 (9-17)
SARS-CoV-2 infection confirmed tests: antigen test n (%)	41 (100)
Critical presentation, n (%)	21 (52,1)
Clinical and intensive support	
Gastrointestinal symptoms, n (%)	35 (85.4)
Mucocutaneous rash, n (%)	31 (75.6)
MIS-C clinical form: Shock syndrome Kawasaki disease, n (%)***	14 (34.1)
Cardiogenic shock, n (%)	17 (68)
Vasoactive inotropic-score at admission/and after 72h from admission median (IQR)	25 (15-93)/70 (22-80)
Oxygen therapy modalities	
*Low flow interfaces* n (%)	8 (19.5)
*Invasive mechanical ventilation* n (%)	22 (53.7)
*Non-invasive mechanical ventilation* n (%)	11 (26.8)
Oxygen index at admission/and after 72h from admission median (IQR)	10.6 (8.4-14.8)/10.3 (6.5-13.9)
PaO_2_/FiO_2_ ratio at admission/and after 72 h from admission median (IQR)	204 (159-337)/113 (75-153)
Ventilator-associated pneumonia, n (%)	9 (42.9)
ADRS, n (%)	20 (48.8)
Multiple organ dysfunction: more than 3 organ involvement, n (%)	26 (63.4)
Renal replacement therapy, n (%)	6 (14.6)
Treatment after 72h from admission, n (%)	
Time to start immunomodulatory therapy, in days, median (IQR)	3.5 (3-5)
Low molecular weight heparin therapy n (%)	35 (85.4)
Methylprednisolone pulse therapy n (%)	27 (65.9)
Intravenous immunoglobulin therapy**** n (%)	37 (90.2)
Outcomes	
Length of stay at hospital, in days, median (IQR)	12 (7-18)
Length of stay at PICU, in days, median (IQR)	9 (5-14)
Oxygen time in days, median (IQR)	6 (4-12)
Mechanical ventilation time in days, median (IQR)	7 (4-12)
Ventilator free days at 28^th^ day, median (IQR)	19 (14-23)
In hospital mortality at 28^th^ day, n (%)	16 (39)
PRISMIV, median (IQR)	19 (10.5-39)
PELOD-2, median (IQR)	12 (8-20)

* 25 children under 5 years old (7/25). ** 16 children over 5 years old (5/16). ADRS, acute distress respiratory syndrome. PRISMIV, pediatric risk of mortality IV. PELOD-2, pediatric organ logistic dysfunction. IQR, interquartile range. *** hemophagocytic lymphohistiocytosis, (HLH):13/41 (31.7%), Kawasaki disease: 8/41 (19.5%), toxic shock syndrome: 3/41 (7.3%); fever of unknown origin: 3/41 (7.3%). ****None of the patients included received IGIV until day 3.

### Comparison of laboratory markers between day 1 and 3

The assessment of non-specific laboratory tests between day 1 and day 3 revealed significant improvement in lymphocytes and platelets as well as in inflammatory markers, such as CRP, ESR and ferritin (**Table 2**). The analysis of oxidative stress markers demonstrated significant differences between Day 1 and Day 3. Levels of TEAC and GSH exhibited a notable upward trend, with TEAC (mmol/L) increasing significantly from 1.67 to 2.06 (p < 0.001) and GSH (µmol/mL) rising from 34 to 37 (p = 0.02). These changes are indicative of enhanced oxidative defense mechanisms and potentially reflect increased metabolic activity during the hospitalization period. Conversely, MDA (mmol/mL) levels decreased significantly from 3.62 to 1.96 (p = 0.002), catalase (U/mg protein) levels declined from 0.042 to 0.035 (p < 0.001), and nitrite (µM/mg protein) levels were markedly reduced from 80.5 to 54.3 (p < 0.001). These reductions suggest a diminishing inflammatory and oxidative state, likely corresponding to clinical improvement and a positive therapeutic response. In contrast, cytokine levels did not exhibit significant differences between Day 1 and Day 3, as demonstrated in **Table 2**.

**Table 2:** Laboratorial characteristics of the cohort.

Characteristics	Hospitalized children with MIS-C (n=41)		
Non-specific laboratorial, median (IQR)	Day 1	Day 3	p value**
**Lactate (mmol/L)**	1.5 (1.2-2.3)	2.2 (1.2-2.4)*	0.655
**Leukocyte/mm^3^**	10,200 (6,800-13,600)	11,500 (7,600-16,000)	0.134
**Lymphocytes/mm^3^**	1,248 (852-1,674)	2,266 (1,248-4,292)	**<0.001**
**Platelets/mm^3^**	127,500 (28,500-317,000)	296,000 (202,000-450,000)	**0.010**
**Hemoglobin (g/dL)**	10.5 (9.8-11.4)	9.8 (9-11.1)	0.164
**INR**	1.46 (1.01-1.48)	1.23 (1.04-1.91)	0.881
**D-Dimer (ng/dL)**	2,014 (1,209-4,125)	1,264 (711-3,861)	0.059
**Fibrinogen (mg/dL)**	248 (148-453)	173 (51-354)	0.761
**C-reactive protein(mg/dL)**	45 (18.3-84.6)	20.2 (6.3-41.2)	**0.002**
**Erythrocyte sedimentation rate (mm/h)**	40 (30-47)	17 (10-54)	**<0.001**
**Ferritin (ng/mL)**	550 (298-954)	314 (107-548)	**0.012**
**Urea (mg/dL)**	27 (20-49)	30 (13-49)	0.143
**Creatinine (mg/dL)**	0.4 (0.3-0.6)	0.3 (0.2-0.5)	0.063
**AST (IU/L)**	48 (36-70)	50 (34-70)	0.677
**ALT (IU/L)**	41 (17-52)	31 (18-71)	0.375
**LDH (IU/L)**	428 (302-685)	470 (306-724)	0.771
**Albumin (g/dL)**	2.7 (2.1-3.0)	2.8 (2.5-3.2)	0.251
**Troponin I (ng/L),**	0.31 (0.06-0.57)	0.2 (0.012-0.4)	0.885
**Creatine phosphokinase-MB fraction (IU/L)**	30 (24-136)	32 (19-52)	0.013
**Creatine phosphokinase (IU/L)**	43 (29-89)	56 (28-297)	0.251
Immunological biomarkers, median (IQR)	Day 1	Day 3	p value**
**IL-17A(pg/mL)**	14.96 (14.78-14.15)	14.42 (14.37-14.9)	0.816
**IFN-γ (pg/mL)**	9.97 (9.68-9.29)	8.31 (8.85-8.77)	0.136
**TNF-α (pg/mL)**	9.58 (9.46-9.99)	9.36 (9.7-9.03)	0.488
**IL-10 (pg/mL)**	11.59 (11.3-11.8)	13.76 (13.37-13.03)	0.118
**IL-6 (pg/mL)**	25.2 (25.45-195.27)	24.35 (24.04-214.4)	0.361
**IL-4 (pg/mL)**	11.58 (11.07-11.22)	9.08 (9.35-9.39)	0.555
**IL-2 (pg/mL)**	9.52 (9.2-9.26)	8.05 (8.2-8.3)	0.079
Inflammatory biomarkers, median (IQR)	Day 1	Day 3	p value**
**TEAC (mmol/L)**	1.67 (1.45-1.88)	2.06 (1.96-2.17)	**<0.001**
**MDA (mmol/m L^-1^)**	3.62 (3.35-3.82)	1.96 (1.66-1.98)	**0.002**
**GSH (µmol/mL)**	34 (35-36)	37 (34-38)	**0.02**
**Catalase (U/mg protein)**	0.042 (0.026-0.056)	0.035 (0.022-0.047)	**<0.001**
**Nitrite in the plasma (µM/mg protein)**	80.5 (70.7-80.2)	54.3 (44.43-54.2)	**<0.001**

*Measured at 24 hours from admission. **Using Wilcoxon signed-ranks test, the bold values refer to a two-tailed p-value less than 0.05. INR, international normalized ratio. AST, alanine transaminase. ALT, aspartate transaminase. LDH, lactate dehydrogenase. IL, interleukins. IFN-γ, interferon‐gamma. TNF-α, tumor necrosis factor alpha. TEAC, Trolox Equivalent Antioxidant Capacity. MDA, malondialdehyde. GSH, glutathione.

### Comparison according to the outcomes

There were 25 survivors and 16 non-survivors. Regarding cytokines, non-survivors presented higher levels of IL-17A, IL-6 and IL-4 compared to survivors on day 1 (p<0.001, p=0.001, and p=0.001, respectively) and day 3 (p<0.001, p=0.002 and p<0.001, respectively). Regarding oxidative stress markers, non-survivors presented higher levels of plasma nitrite in both days (p<0.001), and lower levels of glutathione reductase on day 3 (p<0.001) compared with survivors, as shown in **Table 3**. The elapsed time between admission and the initiation of immunomodulatory therapy did not reveal difference (p=0.575) between non-survivors (median 3.5 days, IQR 3.2-4.5) and survivors (median 3.5 days, IQR 3.6-5.2).

**Table 3:** Serum biomarkers, according to in-hospital mortality.

Characteristics	Survivors (n=25)	Non-survivors (n=16)	p value*
**IL-17A, day 1 (pg/mL)**	5.9 (4.75-13.32)	23.74 (18.67- 44.26)	**<0.001**
**IL-17A, day 3 (pg/mL)**	9.58 (7.37- 15.25)	29.73 (16.53-48.7)	**<0.001**
**IFN-γ, day 1 (pg/mL)**	8.78 (8.24- 11.29)	11.36 (9.65-12.73)	0.054
**IFN-γ, day 3 (pg/mL)**	8.49 (8.26-9.05)	8.04 (6.74-8.36)	0.118
**TNF-α, day 1 (pg/mL)**	8.82 (6.47-9.7)	11.42 (10.36-15.85)	0.004
**TNF-α, day 3 (pg/mL)**	8.44 (6.73-10.44)	10.23 (7.31-11.85)	0.19
**IL-10, day 1 (pg/mL)**	11.1 (8.01-12.25)	14.15 (11.59-19.2)	0.006
**IL-10, day 3 (pg/mL)**	12.24 (9.99-17.61)	15.49 (11.2-19.92)	0.356
**IL-6, day 1 (pg/mL)**	10.93 (7.09-15.99)	18.23 (15.83-24.23)	**0.001**
**IL-6, day 3 (pg/mL)**	10.82 (7.26-17.29)	28.31 (14.35-119.08)	**0.002**
**IL-4, day 1 (pg/mL)**	9.11 (7- 11.77)	12.76 (11.83-16.39)	**0.001**
**IL-4, day 3 (pg/mL)**	5.61 (5.02-9.08)	15.29 (13.5-23.85)	**<0.001**
**IL-2, day 1 (pg/mL)**	9.17 (7.26-10.25)	10.91 (9.21-13.18)	0.01
**IL-2, day 3 (pg/mL)**	8.17 (7.2-8.84)	8.04 (7.12-10.16)	0.873
**TEAC, day 1 (mmol/L)**	1.59 (1.39-1.84)	1.74 (1.48-2.06)	0.285
**TEAC 3, day 3 (mmol/L)**	2.07 (1.98-2.19)	1.98 (1.74- 2.17)	0.153
**Malondialdehyde, day 1 (mmol/m L^-1^)**	2.9 (1.95-4.63)	4 (1.68-5.27)	0.415
**Malondialdehyde, day 3 (mmol/m L^-1^)**	1.85 (1.33-2.62)	2.31 (1.93-3.53)	0.025
**Glutathione, day 1 (µmol/mL)**	42 (31-45)	43 (33-46)	0.378
**Glutathione, day 3 (µmol/mL)**	44 (42-45)	33 (31-38)	**<0.001**
**Catalase, day 1 (U/mg protein)**	0.056 (0.033- 0.061)	0.031 (0.021-0.042)	0.007
**Catalase, day 3 (U/mg protein)**	0.042 (0.028-0.051)	0.025 (0.017-0.035)	0.007
**Nitrite, day 1 (µM/mg protein)**	69.4 (61.5-81.2)	88.8 (83.1-96.7)	**<0.001**
**Nitrite, day 3 (µM/mg protein)**	46.8 (41.5-54.8)	59.9 (56.1-65.3)	**<0.001**

*The bold values show the Bonferroni method (p=0.0021).

Cardiogenic shock was present in 17 (41%) patients; levels of IL-4 were significantly higher on day 3 in the group with cardiogenic shock compared to the group without cardiogenic shock (p=0.001), with no other differences among the other inflammatory biomarkers (**Table 4**).

**Table 4:** Serum biomarkers, according to the outcome subgroups.

Characteristics	Without cardiogenic shock (n=24)	With cardiogenic shock (n=17)	p value*	LOS PICU > 7 days (n=23)	LOS PICU ≤ 7 days (n=18)	p value*
**IL-17A, day 1 (pg/mL)**	10.94 (5.16-16.37)	22.32 (16.27-29.57)	0.004	19.54 (5.9-27.78)	11.72 (4.61-20.92)	0.068
**IL-17A, day 3 (pg/mL)**	11.4 (7.73-15.58)	17.57 (14.35-44)	0.018	15.59 (11.35-40.24)	11.59 (7.74-16.33)	0.07
**IFN-γ, day 1 (pg/mL)**	9.00 (8.34-11.56)	10.34 (9.28-11.03)	0.261	9.72 (8.68-11.03)	10.04 (8.51-13.87)	0.969
**IFN-γ, day 3 (pg/mL)**	8.4 (7.01-9.24)	8.31 (6.81-8.36)	0.434	8.31 (6.85-9.43)	8.36 (6.81-8.68)	0.742
**TNF-α, day 1 (pg/mL)**	8.93 (7.27-11.82)	10.72 (9.11-13.09)	0.255	11.08 (6.8-14.96)	8.99 (7.46-10.12)	0.156
**TNF-α, day 3 (pg/mL)**	8.37 (6.65-10.55)	10.44 (7.61-11.7)	0.138	9.74 (7.01- 11.11)	8.86 (6.33-10.81)	0.495
**IL-10, day 1 (pg/mL)**	11.21 (8.58-12.67)	12.88 (11.54-18.64)	0.066	11.63 (10.48-16.05)	11.5 (9.07-14.7)	0.618
**IL-10, day 3 (pg/mL)**	12.91 (9.21-18.08)	16.58 (11.74-25.26)	0.19	16.58 (10.37-20.81)	12.13 (9.51-6.11)	0.248
**IL-6, day 1(pg/mL)**	12.08 (7.7-15.72)	18.47 (16.46-24.9)	0.004	16.46 (12.31-23.11)	11.27 (7.09-17.25)	0.088
**IL-6, day 3 (pg/mL)**	11.04 (8.26-21.92)	23.44 (13.48-42.28)	0.06	26.56 (12.93-78.73)	10.79 (7.26-13.48)	0.005
**IL-4, day 1 (pg/mL)**	9.43 (6.82-12.47)	12.4 (11.15-16.72)	0.009	11.77 (9.22-12.89)	9.43 (8.02-15.19)	0.655
**IL-4, day 3 (pg/mL)**	5.66 (5.03-10.52)	16.49 (13.01-22.64)	**0.001**	13.98 (5.61-17.03)	5.84 (5.28-9.44)	0.042
**IL-2, day 1 (pg/mL)**	9.27 (7.27-10.92)	10.79 (8.76-11.29)	0.157	9.52 (8.63-13.1)	9.53 (7.26-10.86)	0.351
**IL-2, day 3 (pg/mL)**	8.11 (7.29-9.25)	8.05 (7.2-9.62)	0.701	8.05 (6.9-9.52)	8.11 (7.7-8.84)	0.713
**TEAC, day 1 (mmol/L)**	1.66 (1.47-1.89)	1.67 (1.38-1.88)	0.751	1.71 (1.47-2.03)	1.56 (1.39-1.83)	0.27
**TEAC, day 3 (mmol/L)**	2.05 (1.97-2.18)	2.062 (1.96-2.17)	0.947	2.09 (1.96-2.19)	2.02 (1.93-2.17)	0.753
**TBARS, day 1 (mmol/m L^-1^)**	3.72 (1.82-4.82)	3.1 (1.96-4.83)	0.937	4.15 (2.62-5.3)	2.64 (1.66-3.86)	0.055
**TBARS, day 3 (mmol/m L^-1^)**	1.95 (1.37-3.07)	1.99 (1.85-3.54)	0.208	2.33 (1.85-3.57)	1.80 (1.32-2.28)	0.01
**GSH, day 1 (µmol/mL)**	35 (34-36)	36 (35-37)	0.801	36 (35-37)	35 (34-36)	0.372
**GSH, day 3 (µmol/mL)**	38 (37-39)	35 (34-37)	0.025	37 (34-38)	38 (37-39)	0.338
**Catalase, day 1 (U/mg protein)**	0.052 (0.031-0.063)	0.034 (0.025-0.043)	0.04	0.042 (0.03-0.056)	0.041 (0.022-0.061)	0.906
**Catalase, day 3 (U/mg protein)**	0.043 (0.025-0.053)	0.028(0.021-0.036)	0.04	0.035 (0.025-0.047)	0.035 (0.018-0.051)	0.906
**NO, day 1 (µM/mg protein)**	67.2 (57.85-83.3)	83.8 (80.2-95.1)	0.005	82.5 (64-92.2)	72 (61.5-83.6)	0.222
**NO, day 3 (µM/mg protein)**	45.36 (39.49-56.28)	56.56 (54.13-64.19)	0.005	55.69 (43.2-62.23)	48.6 (41.513- 56.43)	0.222

*The bold values show the Bonferroni method (p=0.0021). LOS - length of stay in hospital.

In relation to the length of stay in PICU, 23 (56%) stayed longer than 7 days; none of the inflammatory biomarkers stood out in relation to this outcome (**Table 4**). We also carried out a comparison between critical and non-critical patients, however the results were similar to the mortality outcome.

### Kaplan-Meyer curves

**Figure 2:**
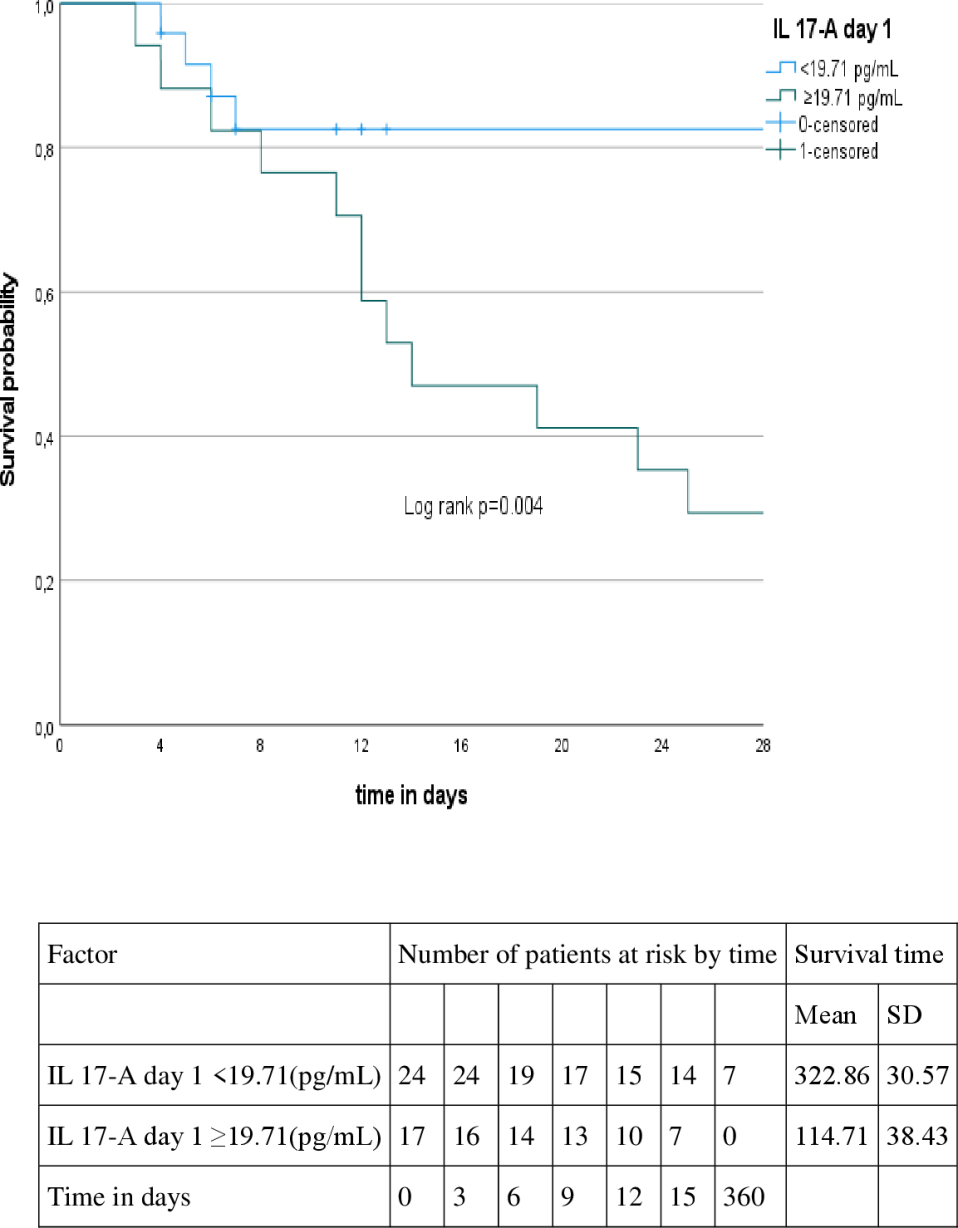
Kaplan-Meyer curve according to the IL 17-A level at day 1.

**Figure 3:**
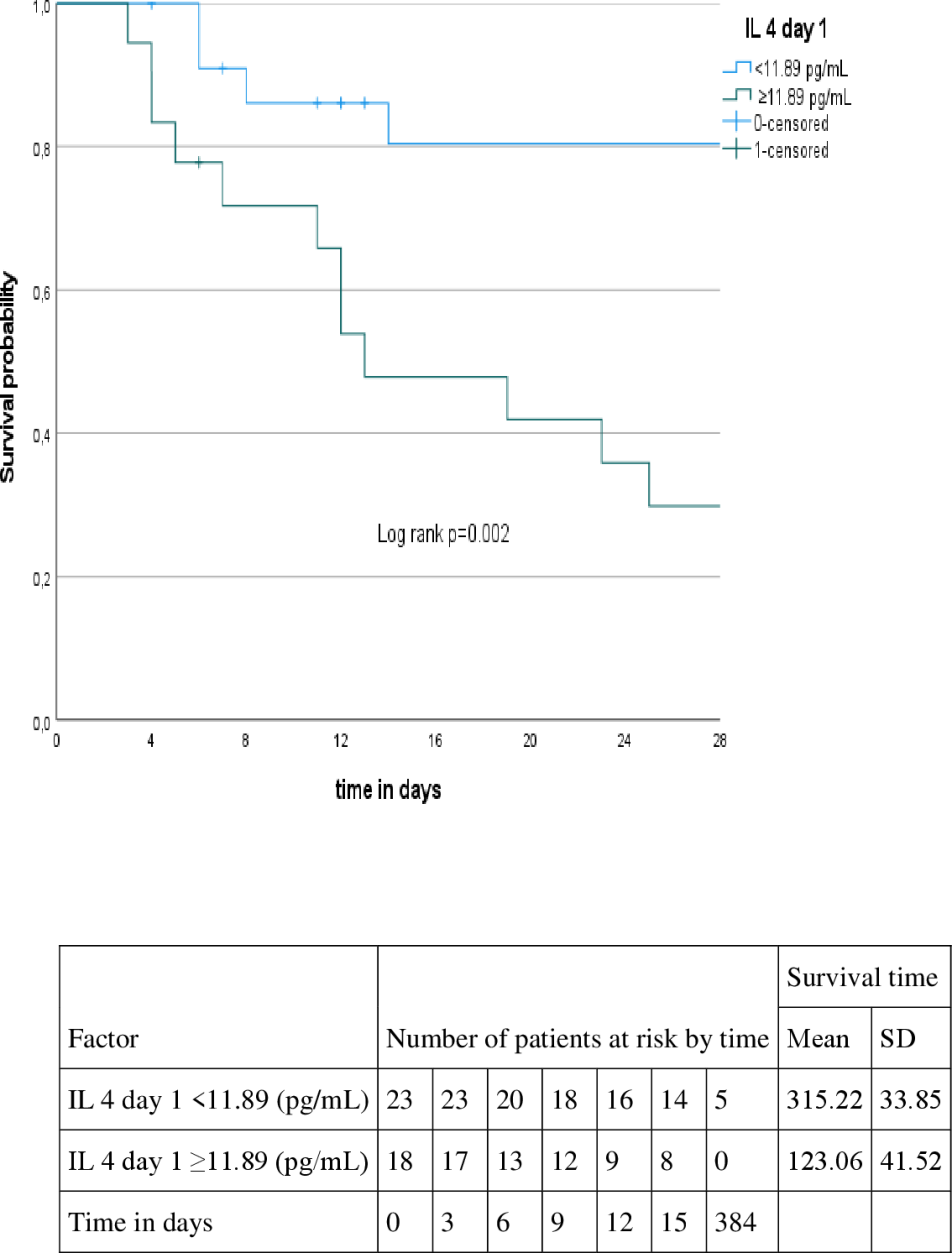
Kaplan-Meyer curve according to the IL 4 level at day 1.

**Figure 4:**
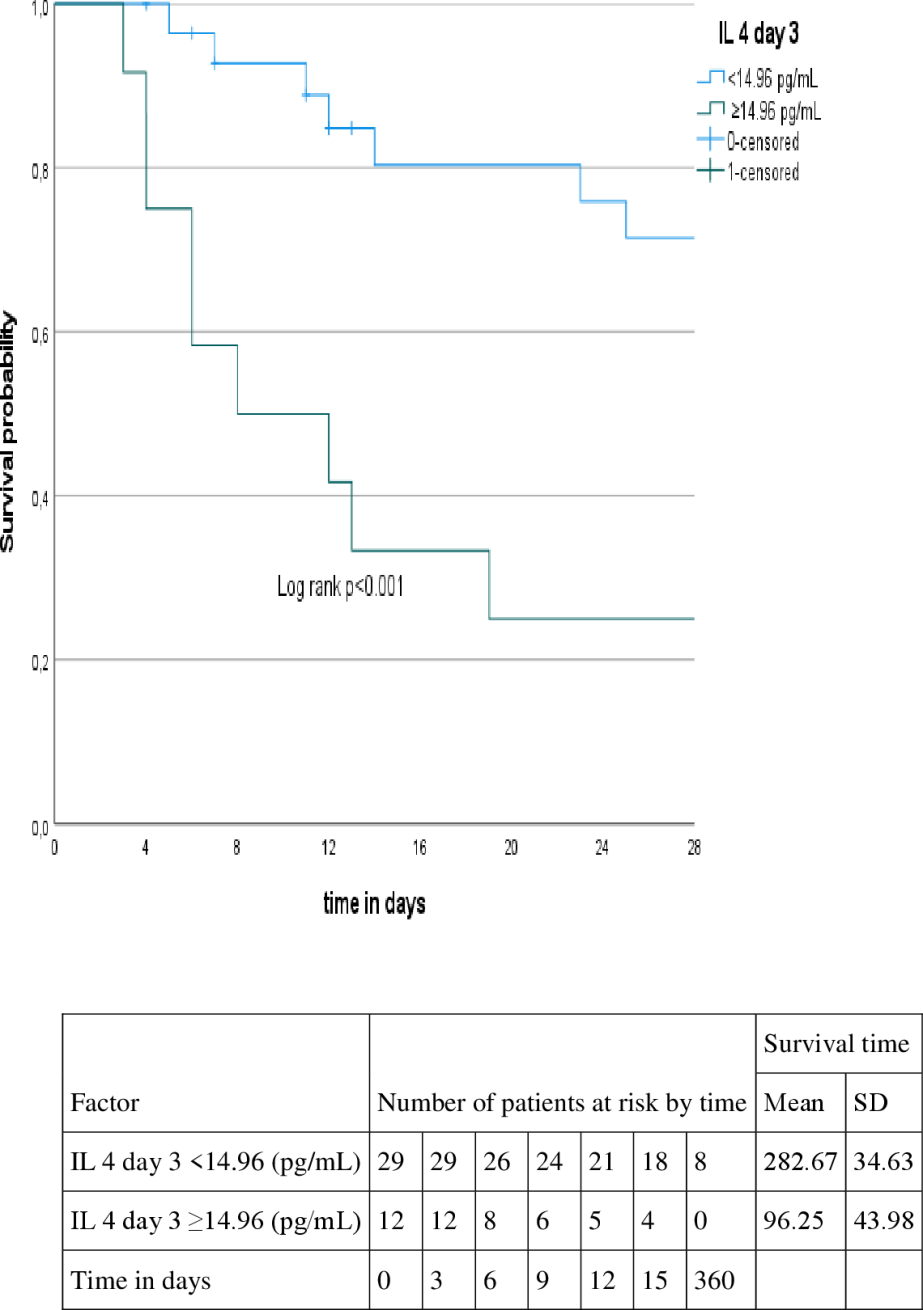
Kaplan-Meyer curve according to the IL 4 level at day 3.

**Figure 5:**
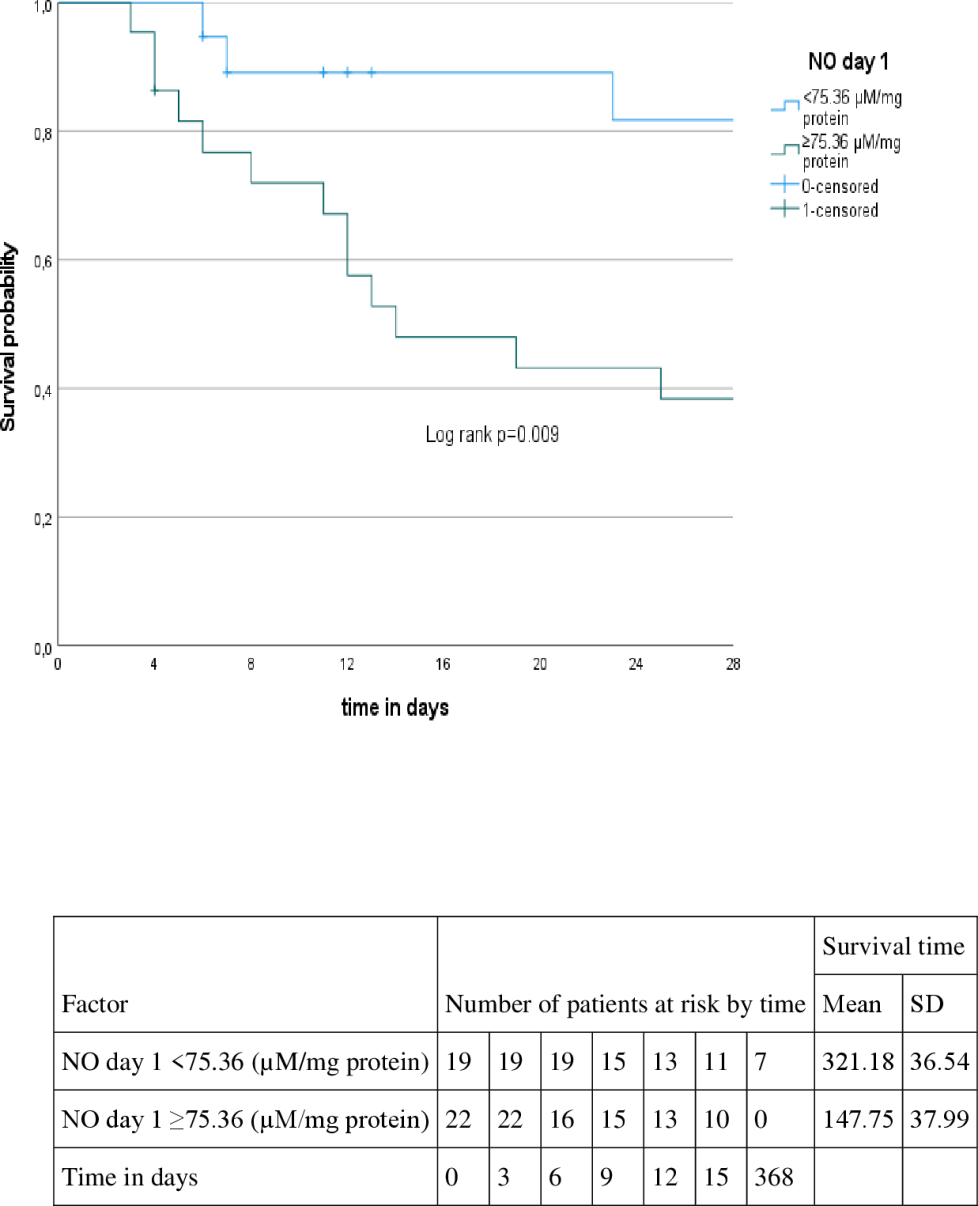
Kaplan-Meyer curve according to the NO level at day 1.

**Figure 6:**
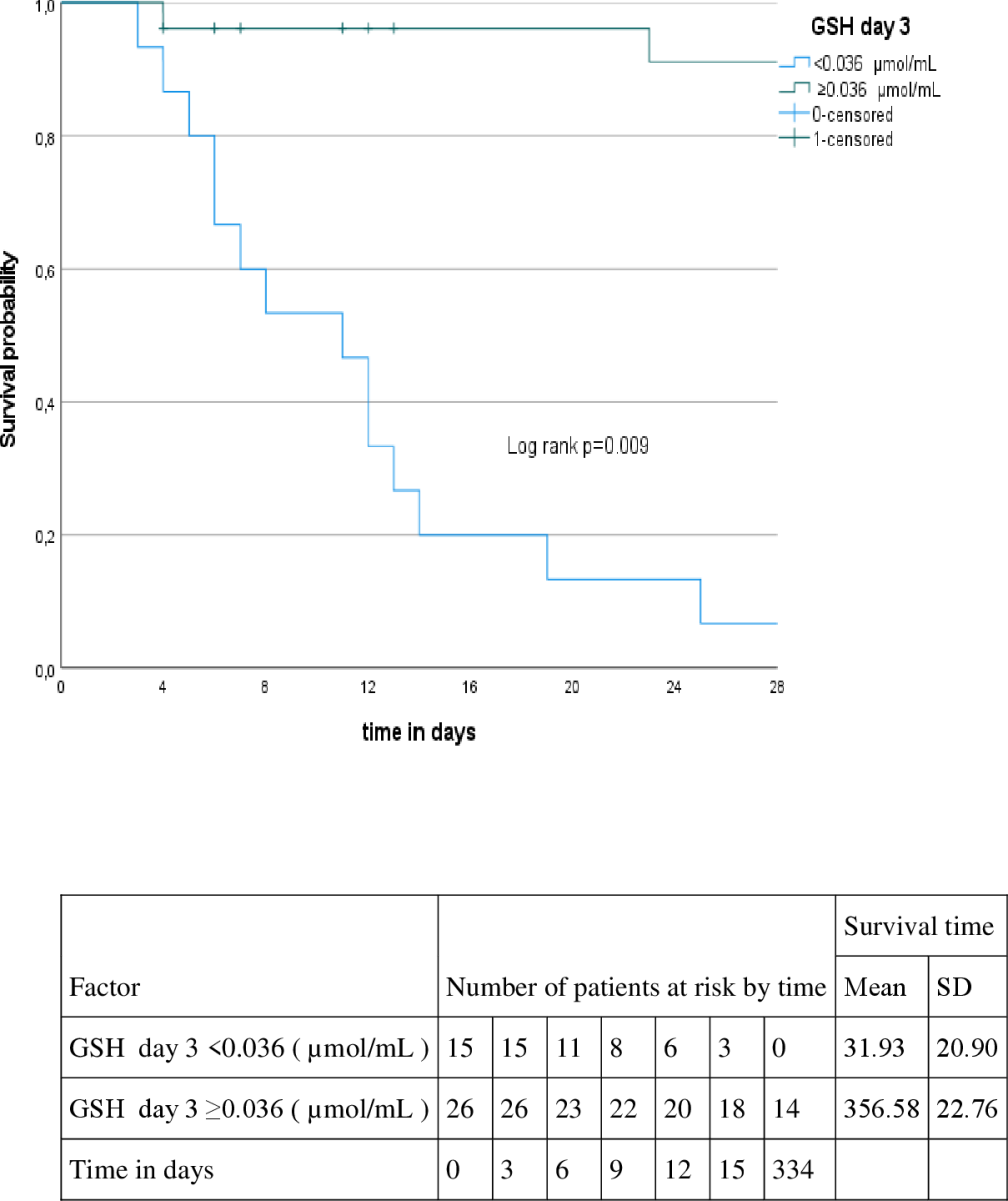
Kaplan-Meyer curve according to the GSH at day 3.

Patients with higher levels of IL-17A (≥ 19.71 pg/mL) on day 1, higher levels of IL-4 (≥ 11.89 pg/mL) on day 1, higher levels of IL-4 (≥14.96 pg/mL) on day 3 and higher levels of NO (≥75.36 µM/mg protein) on day 1 presented shorter survival time (p=0.004, p=0.002, p=0.001, p=0.009, respectively). On the other hand, patients with lower levels of GSH (< 0.036 µmol/mL) on day 3 (p=0.009), presented shorter survival time, as showed in **Figures 2**–**6**.

### Multivariate analysis

After multivariate analysis, IL-17A was the only factor associated with shorter survival time on day 1 either in crude (*HR* 1.03, CI95%1.004-1.057, p=0.022) or adjusted models (*HR* 1.043, CI95%1.013-1.075, p=0.012) as shown in **Table 5**.

**Table 5:** Cox regression analysis for associated factors to in-hospital mortality at 28th day.

Variable	Unadjusted model						Adjusted model*					
	Univariate		*p* value	Multivariate		*p* value	Univariate		*p* value	Multivariate		*p* value
	*HR*	CI95%		*HR*	CI95%		*HR*	CI95%		*HR*	CI95%	
**IL-17 A, day 1 each point up, mean 19.71 pg/mL**	1.03	1.02-1.05	**<0.001**	1.03	1.04-1.06	**0.022**	1.05	1.01-1.07	**0.012**	1.21	1.02-1.46	**0.048**
**IL-4, day 1 each point up, mean 11.89 pg/mL**	1.139	1.05-1.22	**0.001**	0.92	0.81-1.05	0.218	1.25	1.05-1.48	**0.010**	1.03	0.99-1.07	0.102
**IL-4, day 3 each point up, mean 14.96 pg/mL**	1.046	1.02-1.06	**<0.001**	1.05	1.01-1.08	0.06	1.07	0.99-1.15	0.10	–	–	–
**GSH, day 3 each point up, mean 0.036 µmol/mL**	0.002	0.001-0.005	**<0.001**	–	–	—–	0.003	0.001-0.007	**0.016**	–	–	–
**NO, day 1 mean 75.36 µM/mg prot**	1.07	1.02-1.11	**0.002**	1.07	1.02-1.12	0.05	1.03	0.97-1.1	0.327	–	–	–

*Adjusted model: We included as a main cofactor, presence of comorbidity and age less than 1 year old. *HR*, hazard ratio. CI, confidential interval. GSH, glutathione reductase. NO, Nitrite. IL 6 day 1 and day 3, and NO day 3. did not meet the proportional hazards requirement for Cox regression and therefore was discarded from the regression. The bold values expressing significance level were set at two-sided p <0.05. We excluded GSH to multivariate regression since the confidential interval was lower than zero, in unadjusted and adjusted model, GSH day 3 mean 0.036 µmol/mL *HR* 0.002, CI95%0.001-0.005, p<0.001 and *HR* 0.003, CI95%0.001-0.007, p=0.016, respectively.

### ROC analysis

**Figure 7:**
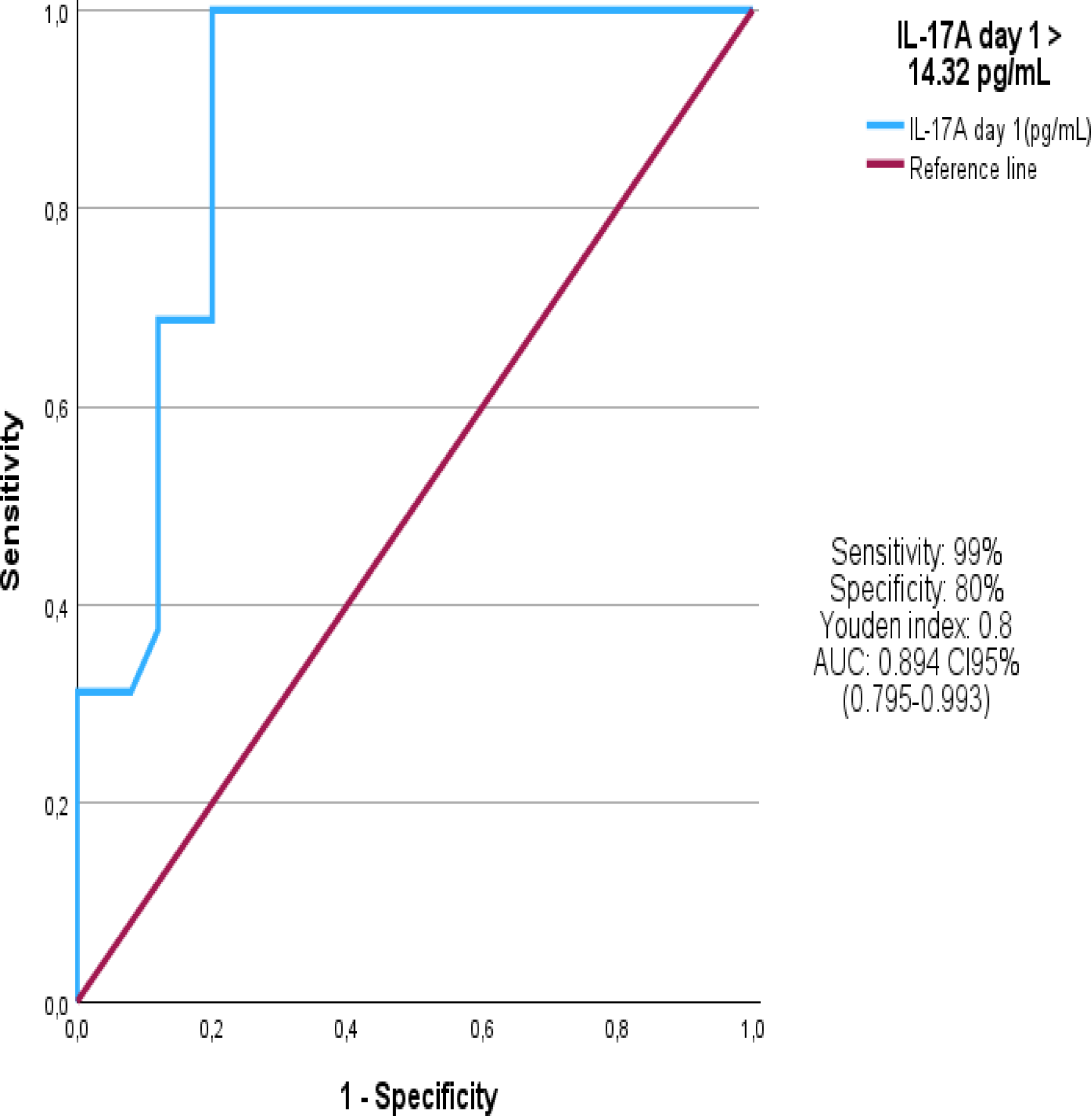
ROC Curve.

As cut-point values of inflammatory biomarkers for mortality have not yet been described in MIS-C patients, we chose to perform ROC analysis to identify the best possible cut-point associated with mortality outcome after we plotted the Kaplan-Meyer curves and performed univariate and multivariate Cox regression. We observed that the cut-off value for the IL-17A was 14.32 pg/ml with sensitivity of 99% and specificity of 80%, as showed in **Figure 7**.

## Discussion

We conducted a study with a substantial cohort of MIS-C patients requiring PICU hospitalization, focusing on clinical and laboratory characteristics, especially inflammatory biomarkers, including cytokines and oxidative stress markers. The findings revealed a high rate of in-hospital mortality, and we examined factors associated with this outcome, such as critical disease, cardiogenic shock, and PICU length of stay. While various inflammatory biomarkers showed associations with mortality, IL-17A was the only factor significantly linked with shorter survival after multivariate analysis, both in crude and adjusted models.

Previous studies have evaluated circulating cytokine profiles in MIS-C patients, but their comparability to our cohort is limited due to differences in sample size, age range, and definitions of COVID-19 and MIS-C, including coinfection presence and immunosuppressant use (6, 11, 43–49). These studies generally reported lower rates of comorbidities, reduced need for intensive care, and lower mortality than observed in our cohort. Despite these differences, prior research has consistently shown elevated inflammatory cytokines, such as IFN-γ, IL-10, IL-6, IL-8, CXCL10, MIP-1α, MIP-1β, TNF-α, and IL-17 in MIS-C patients compared to healthy controls and other pediatric COVID-19 cases (6, 11, 43–49). Nonetheless, a definitive cytokine signature for MIS-C has yet to emerge, and these studies did not examine mortality predictors.

Our findings align with studies on pediatric inflammatory biomarkers (43–49) and those from adult cohorts (50), which suggest that patients requiring PICU admission show significantly elevated IL-6, IL-10, and TNF-α levels and reduced lymphocyte counts. Additionally, elevated CRP, ESR, troponin, D-dimer, ferritin, and a tendency towards thrombocytopenia indicate acute inflammation and potential for organ dysfunction. Troponin elevation suggests myocardial injury, while high fibrinogen, elevated D-dimer, and low platelet counts in the acute phase indicate a procoagulant state and heightened risk of organ dysfunction (6, 20).

This study documented hyperinflammation marked by moderate hyperferritinemia and elevated CRP levels. Previous studies have reported even higher hyperferritinemia levels, which may differ due to our cohort’s high proportion of malnutrition, potentially affecting ferritin expression (6–11, 51, 52). Non-survivors in our cohort exhibited elevated cytokine levels (IL-2, IFN-γ, TNF-α, IL-10) at admission and increased IL-17A, IL-6, and IL-4 levels on days 1 and 3. Notably, IL-17A levels were associated with mortality when adjusted for comorbidity and age under one year. Persistently elevated IFN-γ, IL-6, and IL-10 in non-survivors also raised the possibility of an association with hemophagocytic lymphohistiocytosis (HLH) (53–60). We observed that elevated plasma IL-17A levels in MIS-C patients correlated with reduced survival time, supporting previous reports of high IL-17A in MIS-C (43–50, 53). However, few studies (19, 60–62) have examined mortality risk factors, and none investigated the relationship between cytokine levels, oxidative stress, and mortality. Our findings suggest that IL-17A may play a role in the pathogenesis of severe MIS-C.

Th17 cells, responsible for producing the IL-17 cytokine family (IL-17A to IL-17F), also include other cells such as γδ T cells, NKT cells, NK cells, neutrophils, and eosinophils in IL-17 production (63). IL-17A and IL-17F levels have been linked to chronic inflammatory diseases, such as psoriasis, multiple sclerosis, systemic lupus erythematosus, rheumatoid arthritis, and juvenile idiopathic arthritis (64–67). Th17 cells are known to recruit neutrophils to sites of inflammation, which may contribute to inflammation and tissue damage in MIS-C, similar to mechanisms observed in acute myocardial infarction and autoimmune diseases (64, 66, 68, 69).

For oxidative stress biomarkers, only NO (on both days) and GSH (on day 3) were statistically significant. High NO levels, associated with increased mortality, may result from heightened nitric oxide production, leading to cardiovascular dysfunction, bioenergetic failure, and cellular toxicity. This association is supported by the frequent cardiogenic shock, high VIS levels, and impaired oxygenation in our cohort. Similar associations have been reported in adult COVID-19 and sepsis cases (69–71). The GSH levels in MIS-C non-survivors in our study matched those reported in severe clinical cases, such as septic shock and severe COVID-19, with reduced levels indicating severe oxidative stress (61, 62, 70–74).

Catalase, crucial for decomposing hydrogen peroxide, is a vital defense against ROS-induced cellular damage. Its low levels in non-survivors suggest a weakened antioxidant defense, potentially increasing susceptibility to oxidative stress and contributing to multiorgan damage in MIS-C (12, 13, 17). Additionally, alterations in TEAC and MDA levels indicate oxidative imbalance, with MDA signifying lipid peroxidation and TEAC reflecting total antioxidant capacity, both of which may be insufficient in critical MIS-C cases (14, 18).

In summary, the interplay between cytokine-induced ROS production and oxidative stress may amplify disease severity. This feedback loop could drive further immune activation and oxidative damage, emphasizing the therapeutic potential of antioxidant or anti-cytokine treatments in MIS-C. These findings support that evaluating both cytokine and oxidative stress biomarkers may improve prognostic accuracy and inform future therapeutic approaches for MIS-C (12–18).

This study highlights the potential pathogenic mechanism in critical MIS-C involving an exaggerated immune response with self-perpetuating inflammation, paralleling autoimmune disease processes. However, further research is needed to confirm this association in MIS-C (11, 45, 47, 69). Although biomarker development faces practical limitations, it remains crucial for predicting diagnosis and mortality in critical patients. Thus, biomarkers with high diagnostic and prognostic value, especially those linked to actionable therapeutic options, are a primary focus in critical care (75).

The COVID-19 pandemic presented a profound health challenge. While MIS-C prevalence and mortality remain low, particularly in developed nations, evidence suggests that MIS-C disproportionately affects Black and Hispanic children, those under one year, and those with comorbidities. Our study corroborates this, identifying increased disease severity and mortality among Brazilian “Pardo” children, possibly due to higher SARS-CoV-2 exposure in socioeconomically disadvantaged regions and potential genetic factors (75–78).

In addressing the diagnostic limitations of this study, several constraints should be acknowledged that may impact the generalizability and depth of our findings. Firstly, as a single-center study, data collection was restricted to one institution, limiting the variability of the population sample and potentially introducing bias specific to regional patient demographics and care practices. The single-center design underscores the need for multicentric studies to validate our results across diverse populations and healthcare settings, which would improve the robustness and applicability of our findings.

Secondly, the absence of immunophenotyping represents a significant limitation, as it restricts our ability to analyze detailed immune cell subpopulations in MIS-C patients. Immunophenotyping would allow for a more nuanced understanding of the specific immune cell profiles and dysfunctions involved in MIS-C, particularly in elucidating roles of T-cell subsets and other critical immune components, which are likely central to MIS-C pathogenesis.

Additionally, methodological variations, particularly in the expression and analysis of cytokine and oxidative stress biomarkers, may influence outcome interpretation. Standardizing biomarker assessment across studies is essential to reduce variability and enhance comparability between findings. Finally, the relatively small patient sample size, a common limitation in MIS-C research, may limit the statistical power of our analyses, reducing the reliability of outcome associations and constraining subgroup analyses.

Together, these limitations highlight the need for future multicentric studies with larger sample sizes, standardized methodologies, and comprehensive immunophenotyping. Such studies are essential to validate the biomarkers identified here and to provide a more detailed understanding of immune and inflammatory processes in MIS-C, ultimately guiding improved diagnostic and therapeutic strategies.

Our study’s strengths include focusing on critically ill MIS-C patients, serial biomarker measurements prior to immunomodulatory therapy, prospective SARS-CoV-2 confirmation, and elimination of epidemiological biases. This study is pioneering within a region marked by healthcare disparities, highlighting a vulnerable subgroup and underscoring the need for targeted interventions. Although overall MIS-C mortality is low, our study shows that younger children with comorbidities face greater mortality risks. Further research should explore the pathophysiological aspects of cytokine and oxidative stress responses in MIS-C, especially the role of IL-17 levels in disease progression. Internationally standardized definitions for MIS-C and neonatal MIS associated with COVID-19 and globally compiled datasets are essential to improve management and guide future trials.

In conclusion, our findings underscore the need to include infants under 12 months in MIS-C research due to their representation in our cohort, contributing valuable insights for all pediatric age groups. This study reinforces the importance of investigating cytokine and oxidative stress biomarkers in MIS-C, as understanding these pathways could facilitate the development of targeted therapies to mitigate severe inflammation and improve patient outcomes. Our research advances understanding of MIS-C by highlighting novel associations between inflammatory biomarkers and mortality. These insights may inform future clinical management strategies, although further studies are essential to validate these findings and investigate therapeutic interventions based on these biomarkers.

## Data Availability

The original contributions presented in the study are included in the article/supplementary material. Further inquiries can be directed to the corresponding author.
